# Stereotactic Radiosurgery for Malignant Melanotic Nerve Sheath Tumors: A Case Series

**DOI:** 10.7759/cureus.112161

**Published:** 2026-07-06

**Authors:** Catelina C Nguyen, Jennifer S Chiang, Erqi Pollom, Elham Rahimy, Laurie Tupper, Melanie H Gephart, Michael Lim, Thomas J Wilson, Steven D. Chang, Scott G Soltys

**Affiliations:** 1 Radiation Oncology, Stanford University School of Medicine, Palo Alto, USA; 2 Neurosurgery, Stanford University School of Medicine, Palo Alto, USA

**Keywords:** adjuvant radiation, malignant melanotic nerve sheath tumor, neurosurgery, radiation effects, radiation oncology neurosurgery, stereotactic radiosurgery

## Abstract

Malignant melanotic nerve sheath tumors (MMNSTs) are rare tumors with uncertain clinical behavior. Standard treatment includes surgical resection; however, the potential benefit of adjuvant radiotherapy remains unclear. We present four patients with MMNSTs who were treated with stereotactic radiosurgery following either partial or gross total resection. The series included two females and two males, ranging in age from 18 to 72 years, with tumors located in the spine or intracranially. At follow-up intervals of 1.6, 1.8, 3.5, and 6.0 years after radiosurgery, all tumors remained controlled, with radiographic stability and no evidence of metastatic disease.

## Introduction

Malignant melanotic nerve sheath tumor (MMNST) is a rare neoplasm, accounting for less than 1% of primary peripheral nerve sheath tumors. Previously known as "melanotic schwannoma," retrospective series have highlighted its malignant potential [1]. The term melanotic schwannoma was officially renamed MMNST in the 2020 World Health Organization (WHO) Classification of Soft Tissue Tumors and the 2021 WHO Classification of Tumors of the Central Nervous System [2,3]. Following its reclassification, MMNST has been more clearly defined and distinguished from its benign counterparts [4] and is no longer considered a variant of conventional benign schwannoma.

The primary treatment for MMNST is surgical resection; however, achieving complete tumor removal without causing substantial morbidity can be challenging because of the frequent infiltration of nerve roots. Furthermore, incomplete resection may be associated with a higher risk of tumor recurrence, as supported by a pooled analysis of 71 cases that demonstrated a trend toward a lower likelihood of recurrence in patients treated with gross total resection (GTR) alone compared with subtotal resection (STR) alone [5]. Some studies suggest that adjuvant therapy, including systemic therapy or radiotherapy, may be beneficial [1,5-9]; however, further investigation is needed to define their efficacy. Stereotactic radiosurgery (SRS) is a standard treatment for schwannomas and other nerve sheath tumors of the brain and spine [10]. Data on the use of SRS for MMNST are limited [6]. In this report, we present four patients with MMNST who were treated with definitive or adjuvant SRS. This study was approved by the institutional review board (IRB).

## Case presentation

Case 1

**Figure 1:**
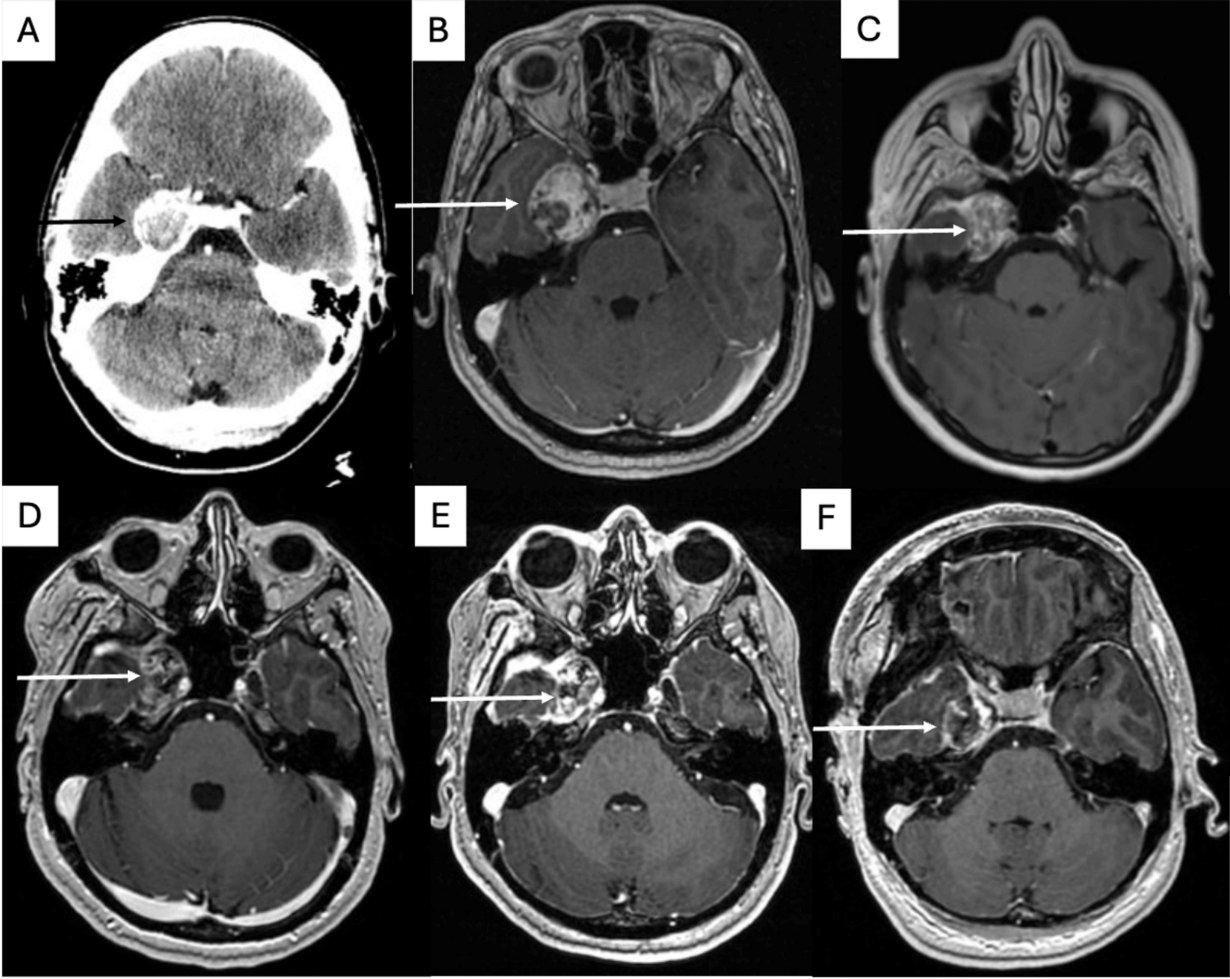
Right cavernous sinus malignant melanotic nerve sheath tumor (Case 1). Axial skull base images, with arrows indicating the tumor, including (A) CT and (B) preoperative MRI demonstrating a T1-hyperintense mass centered in the middle cranial fossa. Postoperative MRIs demonstrate (C) residual tumor one day after surgery, (D) tumor extent immediately prior to SRS, (E) the irradiated tumor at the first follow-up, two months after SRS, and (F) stable residual disease 3.5 years following SRS. SRS: stereotactic radiosurgery.

**Figure 2:**
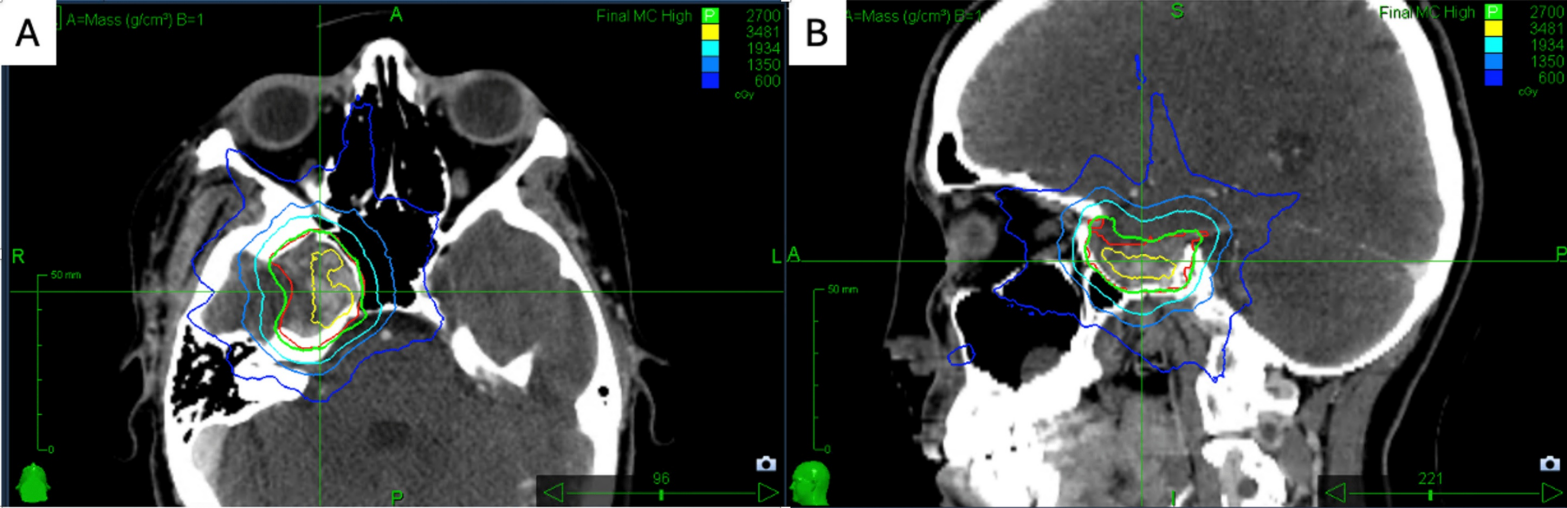
Stereotactic radiosurgery plan for right cavernous sinus malignant melanotic nerve sheath tumor (Case 1). (A) Axial and (B) sagittal views of the clinical target volume (CTV; red contour). The CTV received 2700 cGy in three fractions, prescribed to the 70% isodose line. The 90%, 50%, 35%, and 16% isodose lines are shown in yellow, cyan, blue, and dark blue, respectively. Key organs at risk included the right optic nerve, optic chiasm, and brainstem, which received maximum doses of 1800 cGy, 1157 cGy, and 2151 cGy, respectively.

A 31-year-old woman with a history of migraine headaches associated with photophobia presented to the emergency department with one week of refractory right-sided headaches and bilateral blurry vision. CT revealed a partially calcified 2.6 × 2.8 × 3.0 cm ovoid mass medial to the right temporal lobe. MRI demonstrated a T1-hyperintense and T2-hypointense extra-axial tumor along the medial wall of the right middle cranial fossa, effacing the right Meckel’s cave and medially displacing the cisternal segment of the right trigeminal nerve (Figure 1A). She underwent craniotomy with subtotal resection, during which the tumor was noted to involve multiple cranial nerves. Pathology confirmed MMNST, with pathogenic mutations in PRKAR1A and TP53. Histologically, there was a proliferation of atypical pigmented epithelioid cells with round to ovoid nuclei, prominent nucleoli, and abundant eosinophilic cytoplasm. Immunohistochemistry was positive for MiTF and MelanA and negative for PRAME. Postoperative MRI demonstrated residual nodularity along the right cavernous sinus and within the foramen ovale (Figure 1B). She underwent SRS three months after surgery. On the post-resection MRI (Figure 1C), the clinical target volume (CTV) was delineated to encompass the preoperative extent of tumor contact with the surrounding dura and nerves, as well as the residual tumor. The CTV was treated with 27 Gy in three daily fractions (Figure 2) prescribed to the 70% isodose line, achieving 98% target coverage and a conformity index of 1.2. The brainstem, optic chiasm, and right optic nerve received maximum point doses of 21.5, 11.6, and 18.0 Gy, respectively. Treatment was well tolerated, with only minimal fatigue, headache, and right facial heaviness. The tumor remained controlled, with a stable size at 3.5 years following treatment (Figure 1F).

Case 2

**Figure 3:**
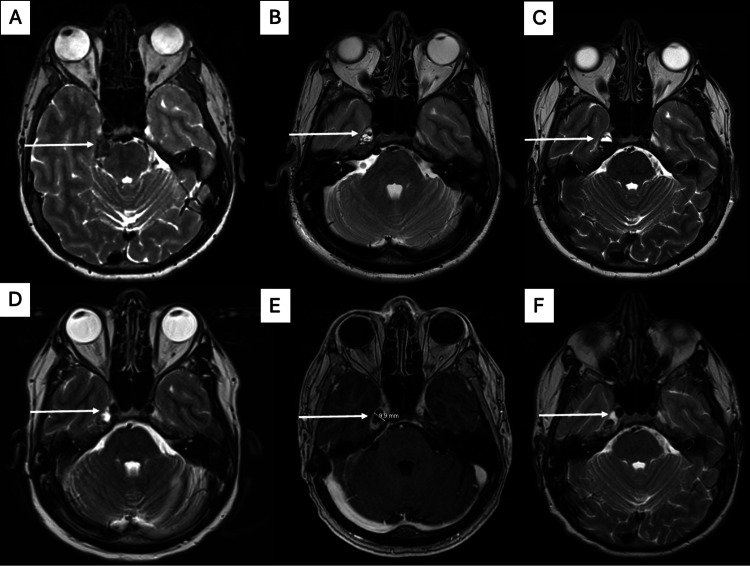
Right Meckel’s cave malignant melanotic nerve sheath tumor (Case 2). Axial T2-weighted MR images, with arrows indicating the tumor, demonstrating (A) preoperative tumor extent, (B) postoperative residual tumor, (C) tumor extent immediately prior to SRS, (D) the irradiated tumor at the first follow-up, 1.6 months after SRS, (E) increased cystic change within the tumor, representing post-treatment tumoral swelling, 10 months after SRS, and (F) stability of the treated tumor, 1.6 years after SRS.

**Figure 4:**
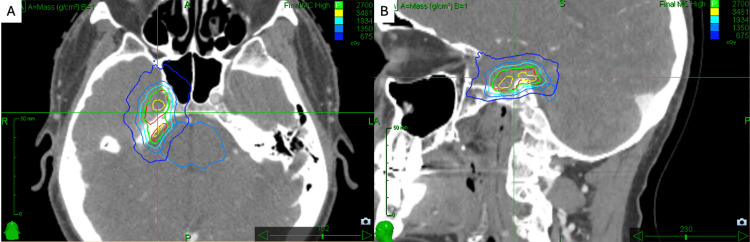
Stereotactic radiosurgery plan for right Meckel’s cave malignant melanotic nerve sheath tumor (Case 2). (A) Axial and (B) sagittal views of the clinical target volume (CTV; red contour). The CTV received 2700 cGy in three fractions, prescribed to the 70% isodose line. The 90%, 50%, 35%, and 17% isodose lines are shown in yellow, cyan, blue, and dark blue, respectively. The brainstem received a maximum dose of 2410 cGy.

A 37-year-old man presented with a one-year history of severe intermittent right facial pain refractory to medication. The pain began as a dull ache in the right temple and progressed to stabbing pain involving the right face and jaw occurring every 45 seconds. Brain MRI demonstrated a T2-hypointense, bilobed tumor involving the cisternal segment of the right trigeminal nerve, measuring 19 × 11 × 11 mm and extending into Meckel’s cave (Figure 3A). He underwent a right craniotomy with subtotal resection, and the pathology was suggestive of a melanotic neoplasm of the nerve. Immunohistochemically, the tumor was positive for SOX10, S100, and MITF. Molecular profiling identified a CTNNA1 D904G mutation and no PRKAR1A mutation. It was noted that a PRKAR1A mutation is not required for the diagnosis of MMNST and that the absence of this mutation does not exclude the diagnosis. Although the final diagnosis was not typical of MMNST, we chose to include this patient in the present series. Postoperative brain MRI demonstrated a decreased size of the cisternal segment component, with a similar size of the component within Meckel’s cave (Figure 3B). For tumor control, the patient underwent SRS three months after surgery. A dose of 27 Gy in three daily fractions was prescribed to the 70% isodose line, with a conformity index of 1.26 (Figure 4). Following SRS, he experienced worsening of pre-existing facial numbness and pain, which progressed despite medications and nerve blocks. MRI performed 1.6 months after SRS demonstrated a tumor measuring 10 × 6 × 10 mm. MRI obtained 10 months after SRS demonstrated an increase in tumor size to 15 × 10 × 13 mm, with a central cystic appearance thought to be consistent with post-SRS swelling commonly observed following treatment of schwannomas (Figure 3E). The decision was made to proceed with repeat imaging in 3-4 months and continue the pain management regimen as needed. Follow-up MRI obtained 1.6 years after radiotherapy demonstrated tumor stability (Figure 3F), at which time the patient's facial pain was controlled with gabapentin and methadone.

Case 3

**Figure 5:**
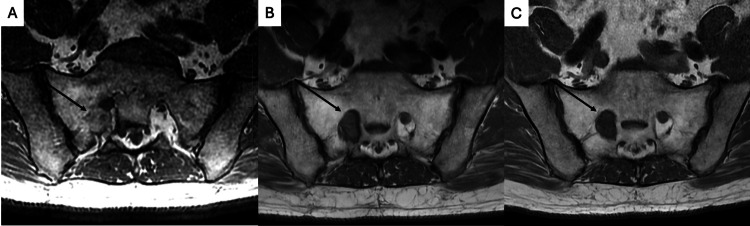
Pre- and post-treatment imaging of the right S1 malignant melanotic nerve sheath tumor (Case 3). Axial pre-contrast T1-weighted MR images, with arrows indicating the tumor, demonstrating the right S1 tumor (A) immediately prior to SRS, (B) 2.6 months after SRS, and (C) 2.4 years after SRS. SRS: stereotactic radiosurgery.

**Figure 6:**
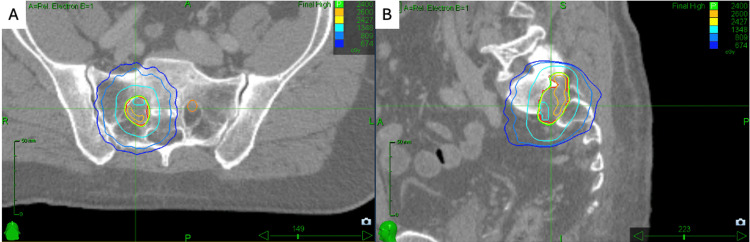
Stereotactic radiosurgery plan for the right S1 malignant melanotic nerve sheath tumor (Case 3). (A) Axial and (B) sagittal views of the clinical target volume (CTV; red contour). The CTV received 2400 cGy in three fractions, prescribed to the 89% isodose line. Note that plan heterogeneity (orange isodose line, 2600 cGy) was intentionally shifted posteriorly, away from the nerve root. The 90%, 50%, 30%, and 25% isodose lines are shown in yellow, cyan, blue, and dark blue, respectively. The cauda equina received a maximum dose of 2021 cGy.

A 72-year-old man presented with a six-month history of mild paresthesias involving the right buttock, posterior thigh, and calf, without other neurological or functional deficits. Spine MRI demonstrated an elongated, enhancing, T1-hyperintense, T2-hypointense mass within the right S1 neural foramen, measuring 20 × 15 mm (Figure 5A). Biopsy confirmed MMNST, with immunohistochemistry positive for SOX10 and HMB45. Given the uncertain aggressiveness of this diagnosis, the patient was informed that surgical resection followed by postoperative radiotherapy, analogous to the treatment approach for sarcoma, would likely maximize tumor control. However, he declined resection because of the potential morbidity associated with an S1 deficit and instead underwent SRS. The T2-weighted MRI sequence aided in tumor delineation. The tumor was treated with 24 Gy in three daily fractions, prescribed to the 89% isodose line (Figure 6). A homogeneous dose distribution (maximum S1 nerve dose, 25.7 Gy) and a slightly lower prescription dose were selected to minimize the risk of nerve toxicity. Target coverage was 97%, with a conformity index of 1.17. The first post-SRS MRI obtained at three months (Figure 5B) demonstrated a stable tumor size (20 × 13 mm), with loss of central enhancement, a finding commonly observed in schwannomas treated with SRS. At his most recent follow-up, 2.4 years after SRS, he reported no new neurological deficits, and the tumor remained stable on MRI, measuring 18 × 12 mm (Figure 5C). 

Case 4

Additionally, we provide an update on a previously published report of an 18-year-old female patient with an S1 MMNST [6]. The patient presented with progressive right sciatic pain for several years that was refractory to physical therapy and medications. MRI demonstrated a T1-hyperintense, T2-hypointense sacral mass. An L5-S1 laminectomy confirmed a right S1 MMNST, with immunohistochemistry positive for S100, SOX10, MITF, HMB45, and MelanA, and a Ki-67 index of <5%. Repeat surgical resection yielded positive margins. To optimize local control, she subsequently underwent SRS. Treatment consisted of 40 Gy in five daily fractions to the pre-resection extent of the tumor and 25 Gy to an approximately 1-cm margin within the spinal canal. The initial report provided 2.5 years of follow-up. We now provide additional follow-up data. At 3.5 years, the tumor remained stable; however, the patient developed right lower extremity numbness, toe weakness, and neuropathic pain that was initially refractory to medication. At 6.0 years, the tumor remained stable on MRI, measuring 4.2 × 2.5 × 1.8 cm. Her sciatic symptoms improved, and she was no longer taking medications. However, she continued to experience foot pain, which was thought to be musculoskeletal rather than neuropathic in origin and remained refractory to treatment with nonsteroidal anti-inflammatory drugs, acetaminophen, gabapentin, and steroid injections. Most recently, the patient was referred for physical therapy, and her response to this intervention is pending. 

## Discussion

MMNST is a rare peripheral nerve sheath tumor that most commonly occurs in the spinal and paraspinal soft tissues [1,5]. Its diagnosis is challenging and requires integration of clinical, radiographic, and histopathologic features to distinguish it from similar entities, including schwannomas, neurofibromas, meningiomas, and melanoma. Clinical presentation is variable and nonspecific, depending on the involved nerve. Characteristic MRI findings include T1 hyperintensity and T2 hypointensity due to melanin's paramagnetic properties, with homogeneous post-contrast enhancement. Histopathologically, MMNST is characterized by spindle and epithelioid cells arranged in interlacing fascicles with marked melanin accumulation within neoplastic cells [11]. Immunohistochemically, MMNSTs typically express neural crest markers (S100, SOX10) and melanotic markers (HMB45, MelanA) and may demonstrate PRKAR1A inactivation, although this alteration is not required for diagnosis [1]. Psammomatous variants account for approximately 40%-50% of melanotic schwannomas [12,13]. Approximately half occur sporadically, whereas the remainder arise in association with Carney complex [12,13]. Carney complex is an autosomal dominant disorder characterized by spotty skin pigmentation, multiple endocrine neoplasms, and myxomas involving the heart, skin, and breast. Although psammomatous features and Carney complex were absent in all patients in our series, the radiographic and immunohistochemical findings were otherwise consistent with MMNST.

Surgical resection remains the primary treatment for MMNST; however, outcomes remain suboptimal. In one systematic review of surgically treated patients, in which 10% underwent STR with adjuvant radiotherapy and 5% underwent GTR with adjuvant radiotherapy, pooled rates of local recurrence, distant metastasis, and mortality were 42%, 27%, and 26%, respectively [5]. These findings underscore the need to explore additional adjuvant strategies. Postoperative management remains poorly defined, with no established guidelines regarding the optimal timing, modality, or indications for adjuvant therapy. Reports describing adjuvant radiotherapy exist; however, available data are limited and largely derived from individual case reports and small case series.

Most reports of adjuvant radiotherapy for MMNST involve conventionally fractionated radiotherapy (Table 1) [5,9,14-16]. Topf et al. reported a left parapharyngeal MMNST treated with adjuvant radiotherapy to a total dose of 60 Gy, although follow-up information was unavailable [9]. Another case described a C7 lesion managed with GTR followed by 55 Gy of radiotherapy, with no evidence of recurrence at 16 months postoperatively [16]. Shields et al. reported two patients with T7 and L5 lesions who underwent STR and GTR, respectively, followed by radiotherapy (dose not reported). Both tumors recurred, at eight months and two years, respectively [15]. Takatori et al. described a patient with an L4 tumor treated with STR and adjuvant radiotherapy (dose not reported) who subsequently developed lung metastases nine months after surgery and died of widespread metastatic disease at 22 months. At autopsy, metastases involved the lungs, spinal cord, bilateral chest wall, and stomach, with no evidence of local recurrence. In a systematic review of 63 patients, six received radiotherapy after STR and three after GTR; among these individuals, four developed local recurrence and four developed metastatic disease [5].

**Table 1:** Clinical, pathologic, and treatment characteristics of reports of MMNST treated with radiotherapy. *Current. IHC: immunohistochemistry, GTR: gross total resection, STR: subtotal resection, RT: radiation, CFRT: conventionally fractionated radiotherapy, SRS: stereotactic radiosurgery, CK: creatine kinase, EMA: epithelial membrane antigen, GFAP: glial fibrillary acidic protein, CD45: cluster of differentiation 34, SMA: smooth muscle actin, NSE: neuron-specific enolase, FU: follow-up, TMB: tumor mutational burden, MMNSTs: malignant melanotic nerve sheath tumors.

Case	Age	Sex	Tumor location	Symptoms	Carney syndrome	Pathology	IHC	Molecular	Resection extent	RT indication	RT technique	RT dose (Gy)	No. of fractions	Recurrence	FU
1 [16]	46	Male	C7 level	Pain and distension in the neck (2 years); numbness in the left hand (1 year)	No	Spindle-shaped tumor cells aligned in intersecting strands; little nuclear polymorphism; normal mitosis and containing large quantities of melanin granules with occasional psammoma bodies and hemorrhages	S100, HMB45, and vimentin positive. CK, EMA, GFAP, factor VIII, CD34, SMA, Syn, and NSE negative	-	GTR	Adjuvant	CFRT	55	-	None	16 months
2 [15]	65	Female	T6-8 level	Mild-thoracic spinal pain	No	Epithelioid and spindle-shaped cells with intracytoplasmic melanotic granules and psammoma bodies	S100 and vimentin positive	-	STR	Adjuvant	-	-	-	Local	8 months
3 [15]	33	Male	L5-S1 level	Low back pain; right L5 radiculopathy	No	Heavily pigmented tumor (limited description available)	-	-	GTR	Adjuvant	-	-	-	Local and distant (lung)	2 years
4 [9]	29	Male	Parapharyngeal	Mild discomfort when sleeping	-	Extensively pigmented melanotic neoplasm with epithelioid cytology and nests and sheets of tumor cells separated by fibrous strands. Areas of tumor necrosis, widespread nuclear atypia with macronucleoli, mitotic activity, and foci of vascular invasion identified. Reticulin pericellular network pattern	S100 positive, Melan-A negative. Ki-67 23%	ATM R250 alteration	GTR	Adjuvant	CFRT	60	-	-	-
4 [14]	39	Male	L4 nerve root	Low back pain; numbness in the left leg	No	Nuclear atypia and mitosis	HMB45 and laminin positive	-	STR	Adjuvant	-	-	-	Local and distant (lung)	9 months (patient expired at 2 years)
5*	31	Female	Skull base	Right-sided headaches and blurred vision (1 week)	No	Atypical pigmented epithelioid cells with round to ovoid nuclei, prominent nucleoli, and abundant eosinophilic cytoplasm admixed with a background of hemorrhage and pigment. Foci of necrosis identified. No mitotic figures seen	MiTF and MelanA positive, PRAME negative. Ki-67 highlights occasional larger nuclei that appear to correspond to pigment-rich MelanA-positive cells	PPKAR1A and TP53 mutations	STR	Adjuvant	SRS	27	3	None	42 months
6*	37	Male	Skull base	Right facial pain (1 year)	No	Spindled to epithelioid cells with enlarged, round to ovoid nuclei and variably prominent nucleoli arranged in loosely cohesive syncytial clusters with a background of increased melanin pigment deposition. No mitotic figures or necrosis seen	SOX10, S100, and MITF positive. Melanotic markers (HMB45, hMART-1 A103, and tyrosine T311) positive. BAP1 with retained nuclear expression. PRAME negative	CTNNA1 D904G mutation. Lack of PPKAR1A mutation. Microsatellite stable. TMB: 1 mut/Mb	STR	Adjuvant	SRS	27	3	None	23 months
7*	72	Male	S1 level	Paresthesias of the right buttock and right lower extremity (6 months)	No	No definite tumor identified on biopsy	SOX10 and S100 positive	-	N/A	Definitive	SRS	24	3	None	22 months
8*	18	Female	S1 level	Progressive sciatic pain (several years)	No	Heavily pigmented lesion comprised of large atypical epithelioid cells growing in sheets. The nuclei are strikingly pleomorphic with pale chromatin, prominent eosinophilic macronucleoli, and voluminous cytoplasm. Rare mitotic figures identified. No psammomatous calcifications seen. Basement membrane deposition around neoplastic cells highlighted by reticulin stain	S100, MITF, HMB45, and MelanA positive. Ki-67 < 5%	No pathogenic variants	STR	Adjuvant	SRS	40	5	None	6 years

Although limited to four patients, this series is, to our knowledge, the largest report of MMNST treated with SRS and the first to include a case managed with definitive SRS without resection [6]. In our cohort, SRS was used in both the adjuvant and definitive settings, expanding the current understanding of its potential therapeutic role. Across all cases, tumors remained controlled at 1.8, 1.9, 3.5, and 6.0 years of follow-up. As commonly observed in schwannomas treated with SRS, post-treatment tumor swelling and loss of central enhancement were observed in two cases. In these patients, post-SRS imaging changes appeared similar to those seen in other nerve sheath tumors. As best characterized in vestibular schwannomas [17], transient tumor enlargement after SRS does not necessarily indicate disease progression and may resolve with longer follow-up. All patients tolerated SRS well. Three of the four patients had pre-existing neuropathic symptoms, including pain or numbness, which may transiently worsen because of post-SRS swelling. Importantly, no patient developed metastatic disease during follow-up. Overall, this series provides preliminary evidence supporting stereotactic radiosurgery as a potential treatment option for patients with MMNST in both adjuvant and definitive settings.

## Conclusions

MMNSTs are rare, diagnostically challenging tumors that require integrated clinical, radiographic, and histopathologic assessment to reliably distinguish them from similar entities. While surgery remains the mainstay of treatment, optimal adjuvant treatment strategies remain ill-defined, with limited evidence available to guide management. In our series, SRS was associated with durable tumor control, demonstrated by stable tumor size on serial imaging, absence of metastatic disease, and minimal treatment-related morbidity. These favorable outcomes were observed in patients treated following gross total or subtotal resection, as well as in one patient treated definitively. Although limited by the small sample size, retrospective design, lack of systematic adverse event grading, and inclusion of only a single patient treated definitively, these findings suggest that SRS may represent a reasonable treatment strategy for selected patients with MMNST. Additional studies with longer-term follow-up are needed to better define the role of SRS in the management of MMNST. We encourage other centers to report outcomes in patients with this rare tumor.
